# Influence of spot sign on the association between rapidly achieving blood pressure reduction and intracerebral haemorrhage outcomes

**DOI:** 10.1093/esj/aakaf024

**Published:** 2026-01-01

**Authors:** João André Sousa, Olalla Pancorbo, Renato Simonetti, Laura Llull, Pilar Coscojuela, Jordi Blasco, Santiago Perez-Hoyos, Álvaro García-Tornel, Noelia Rodriguez-Villatoro, Federica Rizzo, Marián Muchada, Inés Bartolomé, Marta Olivé-Gadea, Jorge Pagola, Marta Rubiera, Sergio Amaro, Yolanda Silva, Luis Prats-Sanchez, Carlos A Molina, João Sargento-Freitas, David Rodriguez-Luna

**Affiliations:** Stroke Research Group, Vall d’Hebron Research Institute, Barcelona, Spain; Department of Neurology, Centro Hospitalar e Universitario de Coimbra, Coimbra, Portugal; Stroke Research Group, Vall d’Hebron Research Institute, Barcelona, Spain; Stroke Research Group, Vall d’Hebron Research Institute, Barcelona, Spain; Department of Neurology, Vall d’Hebron University Hospital, Barcelona, Spain; Department of Neuroscience, Comprehensive Stroke Center, Hospital Clínic, Barcelona, Spain; Department of Neuroradiology, Vall d’Hebron University Hospital, Barcelona, Spain; Department of Interventional Neuroradiology, CDI, Hospital Clínic, Barcelona, Spain; Statistics and Bioinformatics Unit, Vall d’Hebron Research Institute, Barcelona, Spain; Stroke Research Group, Vall d’Hebron Research Institute, Barcelona, Spain; Department of Neurology, Vall d’Hebron University Hospital, Barcelona, Spain; Stroke Research Group, Vall d’Hebron Research Institute, Barcelona, Spain; Department of Neurology, Vall d’Hebron University Hospital, Barcelona, Spain; Stroke Research Group, Vall d’Hebron Research Institute, Barcelona, Spain; Department of Neurology, Vall d’Hebron University Hospital, Barcelona, Spain; Stroke Research Group, Vall d’Hebron Research Institute, Barcelona, Spain; Department of Neurology, Vall d’Hebron University Hospital, Barcelona, Spain; Department of Neuroscience, Comprehensive Stroke Center, Hospital Clínic, Barcelona, Spain; Stroke Research Group, Vall d’Hebron Research Institute, Barcelona, Spain; Department of Neurology, Vall d’Hebron University Hospital, Barcelona, Spain; Stroke Research Group, Vall d’Hebron Research Institute, Barcelona, Spain; Department of Neurology, Vall d’Hebron University Hospital, Barcelona, Spain; Stroke Research Group, Vall d’Hebron Research Institute, Barcelona, Spain; Department of Neurology, Vall d’Hebron University Hospital, Barcelona, Spain; Department of Neuroscience, Comprehensive Stroke Center, Hospital Clínic, Barcelona, Spain; Department of Neurology, Hospital Universitari Dr. Josep Trueta, Girona, Spain; Department of Neurology, Hospital de la Santa Creu i Sant Pau, Barcelona, Spain; Stroke Research Group, Vall d’Hebron Research Institute, Barcelona, Spain; Department of Neurology, Vall d’Hebron University Hospital, Barcelona, Spain; Department of Neurology, Centro Hospitalar e Universitario de Coimbra, Coimbra, Portugal; Stroke Research Group, Vall d’Hebron Research Institute, Barcelona, Spain; Department of Neurology, Vall d’Hebron University Hospital, Barcelona, Spain

**Keywords:** blood pressure, cerebral haemorrhage, computed tomography angiography, stroke, treatment outcome

## Abstract

**Introduction:**

Patients with a CTA spot sign could benefit more from interventions to limit ICH expansion. We evaluated whether its presence modifies the association between systolic blood pressure (SBP) reduction and ICH outcomes.

**Patients and methods:**

A prospective study of patients with ICH < 6 hours and SBP ≥ 150 mmHg at 2 Comprehensive Stroke Centers in Barcelona over 4.5 years. Patients underwent multiphase CTA (arterial, peak venous and late venous phases) and received treatment targeting SBP ≤ 140 mmHg ≤ 60 minutes. We assessed independent associations and interaction of achieving SBP target ≤ 60 minutes and spot sign status (arterial, or secondarily any phase) with hematoma expansion (>6 mL or > 33%) at 24 hours (primary outcome) and 90-day mRS.

**Results:**

Among 207 patients (mean age 71 ± 13.2 years, 134 [64.7%] male), 67 (32.4%) presented an arterial spot sign and 122 (58.9%) achieved SBP target ≤ 60 minutes. Target rates were similar with and without arterial spot sign (38 [56.7%] vs 84 [60.0%], *P* = .653). Hematoma expansion occurred in 46/177 (26.0%), and median 90-day mRS was 4 (2–5). Arterial spot sign and SBP target ≤ 60 minutes were independently associated with hematoma expansion (adjusted odds ratio [aOR] 4.07; 95% CI, 1.74–9.89 and aOR 0.27; 95% CI, 0.11–0.64) and 90-day mRS (aOR 2.23; 95% CI, 1.23–4.07 and aOR 0.43; 95% CI, 0.24–0.76), with no interaction between them (*P* = .575 and *P* = .187, respectively). Similar results were observed considering spot sign in any multiphase CTA phase.

**Conclusion:**

The association between rapidly achieving SBP reduction and ICH outcomes appears neither dependent on nor modified by spot sign status.

## Graphical Abstract

**Graphical Abstract:**
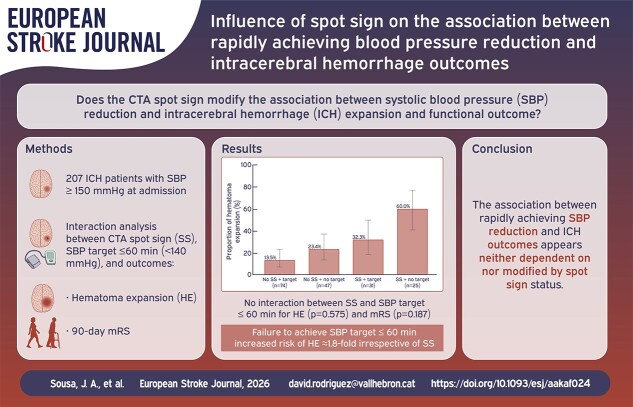

## Introduction

Elevated blood pressure (BP) is frequent in acute ICH and increases the risk of hematoma expansion and poor outcomes.^1^ Rapid BP lowering to an intensive systolic blood pressure (SBP) target < 140 mmHg improves functional outcomes when implemented as part of an acute care bundle,^2^ likely by limiting hematoma expansion.^3^ However, rapidly achieving this BP reduction can be challenging,^4^^,^^5^ and its benefit may be limited, or even harmful, in certain cases,^1^ highlighting the need to identify patients who benefit most from early BP-lowering interventions.

The CTA spot sign is a strong predictor of hematoma expansion,^6^ particularly when identified during the arterial phase of multiphase CTA (arterial spot sign).^7^^,^^8^ This has prompted the hypothesis that spot sign-positive patients may benefit more from interventions limiting hematoma expansion, including hemostatic or BP-lowering therapies.^9–12^ A secondary analysis of Antihypertensive Treatment of Acute Cerebral Hemorrhage II (ATACH-II) patients who underwent CTA did not observe that the association between BP lowering and ICH outcomes depended on spot sign presence.^12^ We hypothesised that rapidly achieving BP reduction reduces hematoma expansion independently of spot sign, but that its presence, particularly in the arterial multiphase CTA phase, may amplify this association.

The main objective was to determine whether the presence of an arterial spot sign modifies the association between rapidly achieving an intensive SBP target and hematoma expansion. Secondary objectives were to evaluate the impact of an arterial spot sign on BP-lowering associations with clinical and functional outcomes, the association of spot sign presence in any multiphase CTA phase, and whether spot sign influences the likelihood of rapidly achieving the SBP target.

## Patients and methods

### Study design

Rapid, Intensive and Sustained BP Lowering in Acute ICH—Spot Sign (RAINSpot) was a prospective, multicenter cohort study embedded within Rapid, Intensive and Sustained BP Lowering in Acute ICH (RAINS), conducted at 2 of the 4 Comprehensive Stroke Centers in Catalonia, Spain, participating in RAINS. RAINS included 312 adults with spontaneous ICH < 6 hours and admission SBP ≥ 150 mmHg, recruited from 1 January 2018 to 30 June 2022, excluding patients with structural or macrovascular causes of ICH, deep coma, planned immediate neurosurgery, pre-established care limitations or contraindication to rapid BP lowering.^3^

### Blood pressure monitoring and management

Patients underwent 24-hour close noninvasive automated BP monitoring and were managed with a standardised protocol targeting rapid (≤60 minutes from antihypertensive treatment initiation), intensive (SBP 110–140 mmHg) and sustained (24-hour stability) BP lowering. SBP target achievement was defined as the first recorded SBP < 140 mmHg.

Antihypertensive treatment was started when SBP ≥ 150 mmHg using an IV bolus immediately followed by continuous infusion, while avoiding the use of intermittent boluses. Urapidil was used as first-line treatment, and labetalol as second-line when SBP target could not be achieved with urapidil infusion.^3^

### Inclusion and exclusion criteria

RAINSpot included RAINS patients from Vall d’Hebron University Hospital and Hospital Clínic, where a standardised multiphase CTA protocol was performed as routine practice or per RAINSpot protocol, respectively. The only additional exclusion criterion for RAINSpot was contraindication to contrast-enhanced CTA (eg, renal impairment or allergy).

### Data collection

At enrollment, demographic, clinical and laboratory data were collected. The first SBP < 140 mmHg after antihypertensive initiation was recorded, with SBP target ≤ 60 minutes as the main explanatory variable. Clinical assessments at 24 hours and 90 days included NIHSS and mRS, respectively, by masked neurologists.

### Neuroimaging acquisition and analyses

Baseline and 24-hour CTs followed standard protocols. Automated multiphase CTA scans with 0.6-mm slices were acquired in 3 sequential phases at fixed post-contrast times, triggered by descending aorta contrast bolus monitoring.^8^ The first phase captured the peak arterial phase (8 seconds after descending aortic attenuation reached 120 Hounsfield units), followed by equilibrium/peak venous and late venous phases at 4 and 15 seconds delays, respectively.^8^

Baseline and follow-up CTs were analysed centrally by an experienced neuroradiologist (J.B.) masked to clinical data for ICH location, intraparenchymal hematoma volume (using semiautomatic Hounsfield-unit threshold-based planimetry in 3D Slicer software) and intraventricular or subarachnoid extension. Multiphase CTAs were reviewed by another experienced neuroradiologist (P.C.) masked to follow-up CTs and clinical data for spot sign, defined as a hyperdense focus within the hematoma.^13^ Spot sign in phase 1 (arterial spot sign), considered a marker of active hemorrhage,^6–8^ was the primary imaging variable, while spot sign in any phase was analysed secondarily.

### Outcomes

The primary outcome was hematoma expansion, defined as an absolute increase in intraparenchymal hematoma volume > 6 mL or a relative increase > 33% on follow-up CT compared to the baseline CT.^3^^,^^14^ Secondary outcomes included early neurological deterioration (increase of ≥ 4 points on the NIHSS or death at 24 hours), the ordinal score on the mRS at 90 days (shift across all categories), independent ambulation (a score of 0–3 on the mRS) at 90 days and functional independence (a score of 0–2 on the mRS) at 90 days.

Patients who underwent surgical hematoma evacuation within the first 24 hours were excluded from the analyses of hematoma expansion and early neurological deterioration, those with a baseline mRS score of ≥ 4 were excluded from the independent ambulation analysis and those with a baseline mRS score of ≥ 3 were excluded from the functional independence analysis.

### Statistical analysis

Complete cases analysis was performed using R 4.5.0. Normality of continuous variables was tested with Shapiro–Wilk. Categorical variables are reported as counts (%) and continuous variables as means ± SD or medians (p25–p75). Intergroup differences were assessed using Pearson *χ*^2^, Student’s *t*, Mann–Whitney *U* or Kruskal–Wallis tests. Multiple logistic and ordinal regression analyses examined independent associations of arterial spot sign and SBP target ≤ 60 minutes with outcomes, adjusting for age, sex, antiplatelet and anticoagulation use, baseline SBP, onset-to-imaging time, ICH volume and intraventricular extension at baseline.^3^^,^^13^ Results are presented as adjusted odds ratios (aORs), 95% CI. The potential for arterial spot sign to modify the association between SBP target ≤ 60 minutes and outcomes was evaluated with an interaction term in the regression models, whose significance was tested using a likelihood ratio test comparing models with and without the term. This interaction was further explored post-hoc in patients who initiated antihypertensive treatment within 2 hours of symptom onset.^15^ Analyses were repeated using spot sign presence in any multiphase CTA phase, and post-hoc sensitivity analyses were performed further adjusting for clinical severity (Glasgow Coma Scale [GCS] and NIHSS scores) and considering time from antihypertensive bolus to SBP target as a continuous variable, as well as considering different ICH expansion definitions.^16^ Absolute and relative risk reductions for hematoma expansion, as well as the number needed to treat (NNT) to prevent one event, were estimated across arterial spot sign and SBP target ≤ 60 minutes strata using observed proportions. Post-hoc pairwise comparisons were performed using Pearson *χ*^2^ for hematoma expansion and Dunn tests for 90-day mRS, applying Bonferroni-adjusted *P* values to control for multiple testing. Two-sided *P* < .05 was considered statistically significant for all tests.

## Results

A total of 207 patients (mean age 71 ± 13.2 years, 134 [64.7%] male) were included (Figure S1). Baseline characteristics are detailed in Table 1. Mean SBP was 177.2 ± 22.6 mmHg, median onset-to-imaging time was 114 (76–201) minutes and median ICH volume was 12.4 (5.6–28.1) mL.

**Table 1:** Baseline characteristics of patients according to the presence of an arterial spot sign and achievement of systolic blood pressure target within 60 minutes

	**All patients**	**Arterial spot sign**			**SBP target ≤ 60 minutes**		
	**(*n* = 207)**	**Yes (*n* = 67)**	**No (*n* = 140)**	*P*-values^*^	**Yes (*n* = 122)**	**No (*n* = 85)**	*P*-values^*^
**Age, mean ± SD, y**	70.7 ± 13.2	72.9 ± 12.3	69.6 ± 13.6	.096	71.8 ± 12.5	69.1 ± 14.2	.142
**Male sex**	134 (64.7)	38 (56.7)	96 (68.6)	.095	78 (63.9)	56 (65.9)	.773
**History of hypertension, *n* (%)**	163 (78.7)	54 (80.6)	109 (77.9)	.652	93 (76.2)	70 (82.4)	.289
**Antihypertensive drugs use, *n* (%)**	139 (67.1)	49 (73.1)	90 (64.3)	.205	84 (68.9)	55 (64.7)	.532
**Antiplatelet drugs use, *n* (%)**	43 (20.8)	16 (23.9)	27 (19.3)	.446	31 (25.4)	12 (14.1)	.048
**Anticoagulant drugs use, *n* (%)**	33 (15.9)	16 (23.9)	17 (12.1)	.031	17 (13.9)	16 (18.8)	.345
**GCS score, median (p25–p75)**	15 (13–15)	14 (12–15)	15 (14–15)	.014	15 (14–15)	15 (13–15)	.081
**NIHSS score, median (p25–p75)**	13 (7–18)	17 (11–20)	12 (6–16)	<.001	12 (6–17)	14 (9–19)	.017
**Systolic BP, mean ± SD, mmHg**	177.2 ± 22.6	179.1 ± 21.9	176.3 ± 22.9	.357	170.5 ± 17.7	186.9 ± 25.2	<.001
**Diastolic BP, mean ± SD, mmHg**	92.5 ± 20.0	91.2 ± 21.0	92.9 ± 19.6	.579	87.4 ± 17.7	99.9 ± 20.9	<.001
**Onset-to-imaging time, median (p25–p75), min**	114 (76–201)	100 (71–139)	125 (84–225)	.002	104 (75–193)	127 (77–152)	.099
**ICH volume, median (p25–p75), mL**	12.4 (5.6–28.1)	25.1 (9.9–35.9)	9.5 (4.5–23.1)	<.001	14.2 (6.4–31.8)	10.7 (4.8–25.9)	.124
**Lobar ICH location, *n* (%)**	55 (26.6)	23 (34.3)	32 (22.9)	.080	34 (27.9)	21 (24.7)	.612
**Intraventricular extension, *n* (%)**	68 (32.9)	24 (35.8)	44 (31.4)	.529	40 (32.8)	28 (32.9)	.981
**Subarachnoid extension, *n* (%)**	40 (19.3)	18 (26.9)	22 (15.7)	.057	29 (23.8)	11 (12.9)	.052

^*^ *P* values determined with Pearson *χ*^2^, Student’s *t* or Mann–Whitney *U* tests.

Abbreviations: BP = blood pressure; GCS = Glasgow Coma Scale.

### Spot sign presence and SBP target achievement

An arterial spot sign was present in 67 (32.4%) patients, and in any multiphase CTA phase in 85 (41.0%). Median time from antihypertensive bolus to SBP target was 59 (33–105) minutes, with 122 (58.9%) patients achieving SBP target ≤ 60 minutes. Variables associated with the presence of an arterial spot sign or achievement of the SBP target ≤ 60 minutes are shown in Table 1. Patients with arterial spot sign were more frequently on anticoagulant therapy (16 [23.9%] vs 17 [12.1%], *P* = .031), had higher NIHSS scores (17 [11–20] vs 12 [6–16], *P* < .001) and lower GCS scores (14 [12–15] vs 15 [14–15], *P* = .014), shorter onset-to-imaging times (100 [71–139] vs 125 [84–225] minutes, *P* = .002), and larger ICH volumes (25.1 [9.9–35.9] vs 9.5 [4.5–23.1] mL, *P* < .001).

Patients with an arterial spot sign achieved SBP target ≤ 60 minutes at rates comparable to those without (38 [56.7%] vs 84 [60.0%], *P* = .653). This finding remained consistent after adjustment for baseline SBP and other relevant confounders (aOR 0.70; 95% CI, 0.35–1.40), and when analysing spot sign in any multiphase CTA phase (aOR 0.60; 95% CI, 0.30–1.17).

### Hematoma expansion

Hematoma expansion occurred in 46 (26.0%) of 177 patients (Figure S1). Among other factors (Table S1), expansion was more frequent in patients with an arterial spot sign (25 [44.6%] vs 21 [17.4%], *P* < .001) and with failure to achieve SBP target ≤ 60 minutes (26 [36.1%] vs 20 [19.0%], *P* = .011). Both associations remained significant in multiple regression analysis (Table 2). No interaction was found between arterial spot sign and SBP target ≤ 60 minutes on hematoma expansion (*P* for interaction = .575), including in 81 (45.8%) patients treated within 2 hours (*P* for interaction = .185) or when evaluating spot sign in any multiphase CTA phase (Table S2). These results were consistent throughout sensitivity analyses (Tables S3 and S4).

**Table 2:** Multiple regression analyses examining the associations of arterial spot sign and SBP target ≤ 60 minutes with outcomes

	**Hematoma expansion**	**Early neurological deterioration**	**mRS at 90 days**	**90-day independent ambulation**	**90-day functional independence**
**Arterial spot sign**	4.07 (1.74–9.89)^*^	2.34 (1.03–5.30)^*^	2.23 (1.23–4.07)^*^	0.40 (0.17–0.94)^*^	0.42 (0.16–1.02)
**SBP target ≤ 60 minutes**	0.27 (0.11–0.64)^*^	0.59 (0.25–1.35)	0.43 (0.24–0.76)^*^	4.55 (1.83–12.25)^*^	3.05 (1.25–7.95)^*^
**Age (per 1-year increase)**	1.07 (1.03–1.11)^*^	1.01 (0.98–1.05)	1.05 (1.03–1.08)^*^	0.92 (0.88–0.96)^*^	0.97 (0.94–1.01)
**Male sex**	0.58 (0.23–1.37)	0.66 (0.26–1.54)	0.69 (0.39–1.21)	1.60 (0.71–3.73)	1.31 (0.57–3.04)
**Antiplatelets**	1.23 (0.44–3.32)	1.17 (0.41–3.17)	0.73 (0.38–1.40)	3.43 (1.21–10.38)^*^	0.70 (0.26–1.83)
**Anticoagulants**	0.96 (0.27–3.21)	2.57 (0.87–7.58)	2.63 (1.18–5.98)^*^	0.21 (0.05–0.79)^*^	0.27 (0.06–1.01)
**Baseline SBP (per 10-mmHg increase)**	1.02 (0.85–1.22)	1.10 (0.92–1.32)	1.08 (0.95–1.22)	0.93 (0.77–1.12)	1.07 (0.89–1.29)
**Onset-to-imaging time (per 10-min increase)**	0.93 (0.87–0.98)^*^	0.95 (0.90–0.99)^*^	0.98 (0.95–1.00)	1.07 (1.02–1.12)^*^	1.05 (1.01–1.10)^*^
**ICH volume (per 10-mL increase)**	0.91 (0.72–1.11)	1.36 (1.16–1.63)^*^	1.44 (1.26–1.67)^*^	0.54 (0.40–0.71)^*^	0.58 (0.42–0.76)^*^
**Intraventricular extension**	0.29 (0.10–0.73)^*^	1.01 (0.42–2.34)	3.06 (1.70–5.58)^*^	0.16 (0.06–0.39)^*^	0.28 (0.11–0.68)^*^

^*^ *P* < .005 (Wald test).

Data are adjusted odds ratio (95% CI). Adjustments were made for age, sex, antiplatelet and anticoagulant use, baseline SBP, onset-to-imaging time, ICH volume and intraventricular extension at baseline.

Abbreviation: SBP = systolic blood pressure.

**Figure 1:**
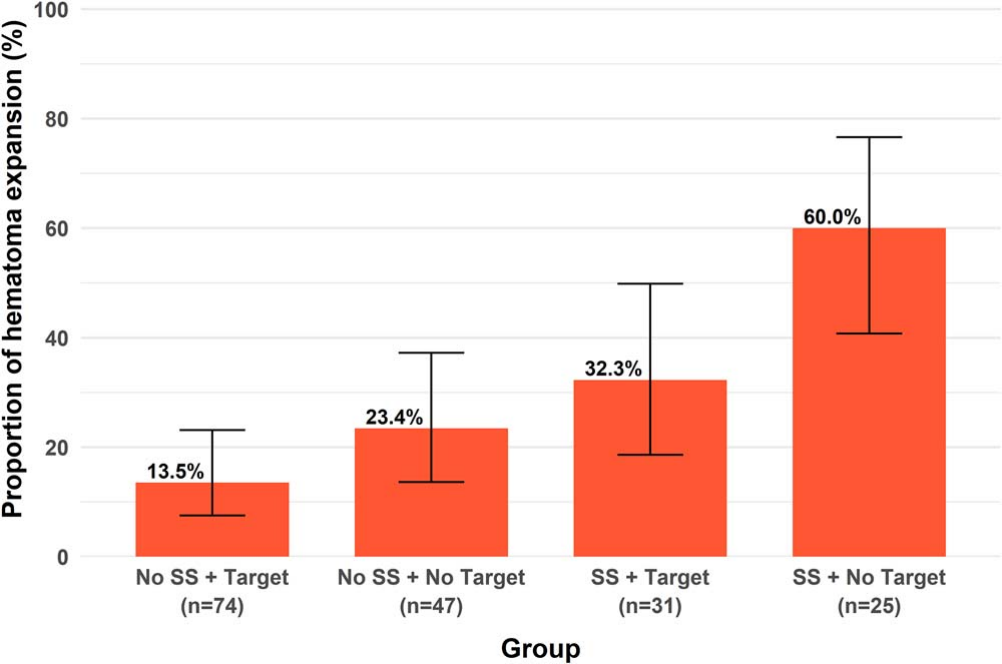
Proportion of patients presenting hematoma expansion stratified by arterial spot sign and achievement of SBP target ≤ 60 minutes. Error bars represent 95% CIs. Abbreviation: SS = spot sign.

Hematoma expansion rates differed significantly across groups stratified by arterial spot sign status and SBP target ≤ 60 minutes (*P* < .001, Figure 1). The presence of an arterial spot sign increased the risk of hematoma expansion ≈ 2.5-fold regardless of SBP target achievement, while failure to achieve SBP target ≤ 60 minutes increased risk ≈ 1.8-fold irrespective of arterial spot sign status. Achieving SBP target ≤ 60 minutes reduced absolute risk by 27.7% in patients with an arterial spot sign and 9.9% in those without, corresponding to relative risk reductions of 46.2% and 42.3%, respectively (Figure 1). The number needed to treat to prevent one hematoma expansion was 3.6 for patients with an arterial spot sign and 10.1 for those without.

Patients with an arterial spot sign who failed to achieve SBP target ≤ 60 minutes had the highest expansion rates (15/25 [60.0%], Figure 1), significantly higher than in patients without an arterial spot sign who achieved (10/74 [13.5%], adjusted *P* < .001) or failed to achieve (11/47 [23.4%], adjusted *P* = .029) SBP target ≤ 60 minutes. No other pairwise comparisons remained significant after correction for multiple testing.

### Clinical and functional outcomes

Patients included in the clinical and functional outcome analyses are shown in Figure S1. Early neurological deterioration occurred in 44 out of 192 (22.9%) patients. At 90 days, the median mRS score was 4 (2–5), with 96 of 201 (47.8%) patients achieving independent ambulation and 62 of 182 (34.1%) patients achieving functional independence.

Both arterial spot sign and failure to achieve SBP target ≤ 60 minutes were associated with poorer outcomes (Table 2). The presence of an arterial spot sign was independently associated with early neurological deterioration, higher mRS scores and lower odds of independent ambulation at 90 days, whereas achieving SBP target ≤ 60 minutes was independently associated with lower mRS scores and higher odds of both independent ambulation and functional independence at 90 days (Table 2). No interaction was observed between arterial spot sign and SBP target ≤ 60 minutes for early neurological deterioration (*P* for interaction = .474), as well as for mRS scores (*P* for interaction = .187), independent ambulation (*P* for interaction = .270) and functional independence (*P* for interaction = .326) at 90 days. Similar results were observed when analysing spot sign in any multiphase CTA phase (Table S2) and in different sensitivity analyses (Tables S3 and S4).

**Figure 2:**
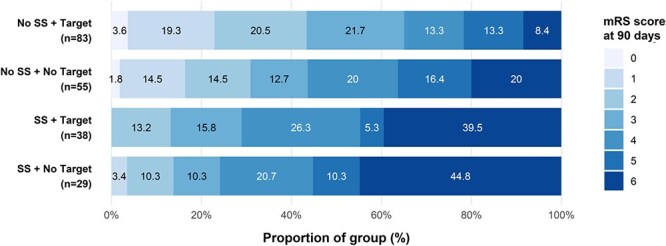
Distribution of mRS scores at 90 days according to arterial spot sign presence and achievement of SBP target ≤ 60 minutes. Abbreviation: SS = spot sign.

The distribution of mRS scores at 90 days differed across 4 groups defined by arterial spot sign status and SBP target ≤ 60 minutes (*P* < .001; Figure 2). Patients without arterial spot sign who achieved SBP target ≤ 60 minutes had significantly better mRS scores than those with arterial spot sign, whether they achieved (adjusted *P* < .001) or failed to achieve (adjusted *P* < .001) SBP target ≤ 60 minutes. No other pairwise comparisons remained significant after adjustment for multiple comparisons.

## Discussion

In this study of patients with acute ICH undergoing multiphase CTA within 6 hours of symptom onset, both failure to achieve the SBP target within 60 minutes and the presence of an arterial spot sign were independently associated with hematoma expansion. Notably, no significant interaction was found between these 2 factors, suggesting that the association between early achievement of an intensive SBP target and hematoma expansion is not substantially modified by the presence of an arterial spot sign, and vice versa. These findings were consistent when considering the presence of a spot sign in any multiphase CTA phase (not limited to the arterial phase), in the subgroup of patients in whom antihypertensive treatment was initiated within 2 hours of symptom onset (although this represents a small subgroup of patients), for functional outcomes, and throughout different sensitivity analyses. Conversely, no associations or inconsistent associations were observed between failure to achieve the SBP target within 60 minutes and spot sign presence with early neurological deterioration, respectively, in the present cohort.

The spot sign is the most robust radiological predictor of hematoma expansion.^6^ While detection in single-phase CTA has been validated in a large, multicenter, prospective study,^17^ multiphase CTA offers improved predictive performance,^8^^,^^18^ and allows differentiation between arterial spot signs, suggestive of active, high-flow bleeding and late/venous spot signs, suggestive of lower-flow or arrested bleeding.^6^^,^^8^ In the present study, early achievement of an intensive SBP target was associated with a lower risk of hematoma expansion and poor functional outcomes regardless of arterial spot sign presence, challenging the notion that spot sign could guide BP-lowering strategies. However, a greater absolute treatment benefit was observed in patients with a spot sign, as indicated by a lower NNT, in line with their higher baseline risk of hematoma expansion. Importantly, our analysis of spot sign presence across all phases of multiphase CTA yielded consistent findings, although associations with outcomes were more robust when restricted to the arterial phase, reinforcing its clinical relevance. Moreover, the likelihood of achieving the SBP target within 60 minutes did not differ by spot sign presence, supporting the feasibility of protocolised early BP management regardless of spot sign status.

Spot sign-based selection has been previously used in trials assessing hemostatic therapies such as recombinant factor VIIa and tranexamic acid. Initial randomised clinical trials testing these treatments showed benefits in reducing hematoma expansion, but results regarding functional outcomes were inconsistent.^19^^,^^20^ To increase the likelihood of demonstrating a potential benefit of hemostatic therapies, a second wave of randomised clinical trials focused on selecting patients at higher risk of hematoma expansion, based on the presence of the spot sign. However, all of them failed to demonstrate radiological or functional benefits,^9–11^ probably because of the relatively small sample sizes and the possibility that most hematoma expansion may have occurred between imaging acquisition and treatment administration.^9^^,^^21^ In line with a substudy of the Factor Seven for Acute Hemorrhagic Stroke (FAST) trial that showed that patients treated within the first 2.5 hours might derive greater benefit from hemostatic therapy with recombinant factor VIIa,^22^ subsequent hemostatic randomised clinical trials have favoured enrolling patients within a narrow time window, such as within the first 2 hours, rather than selecting them based on the presence of the spot sign.^23^^,^^24^ However, 2 recent analyses, an individual patient meta-analysis and a pooled analysis of individual patient data from randomised clinical trials with tranexamic acid, have suggested a potential association in spot sign positive patients treated within 4.5 hours and within 2 hours of onset, respectively,^25^^,^^26^ underscoring the need for further investigation into the interplay between timing, spot sign status and response to hemostatic treatment.

In contrast to hemostatic therapies, no spot sign-based selection randomised clinical trial has been conducted to assess intensive BP reduction. A secondary analysis of the ATACH-II trial, limited to the 13.3% of participants who underwent a single-phase CTA, did not observe that the association between BP lowering and ICH outcomes depended on the presence of the spot sign.^12^ The present study confirms and expands this finding in a larger dataset using a standardised multiphase CTA with fixed timing for image acquisitions. No dependence or modification by spot sign status was observed in the association between BP reduction and outcomes, even when restricted to the arterial phase. Thus, the favourable association of BP reduction with outcomes may be more closely related to timing, as observed in previous studies,^3^^,^^27^ than to the presence of the spot sign. Although the ATACH-II trial failed to demonstrate a beneficial effect of intensive BP reduction on ICH outcomes,^28^ a post-hoc exploratory analysis showed reduced hematoma expansion and improved functional outcome in patients with antihypertensive treatment initiation within 2 hours of symptom onset.^15^ In the present study, however, the association between BP reduction and hematoma expansion or functional outcomes did not appear to depend on or be modified by spot sign status in the subgroup of patients who initiated antihypertensive treatment within 2 hours of symptom onset, even though shorter times enhance the predictive value of the spot sign.^6^

Strengths of this study include prospective, standardised assessment of BP, multiphase CTA spot sign, hematoma expansion and outcomes, with masked evaluation. Limitations include its observational design, which precludes causal inference; a potential reverse causality, as patients with larger baseline hematomas, who are inherently at higher risk of hematoma expansion,^1^ may also have been less likely to achieve rapid BP control; given that the RAINS study was powered for its primary endpoint, the sample size in this embedded study was possibly insufficient to detect modest but clinically relevant differences or interactions between the spot sign and BP lowering, particularly in the subgroup treated within 2 hours; modest sensitivity of the spot sign, which may have limited its observed influence on the association of BP target achievement with ICH outcomes^6^; the definition of hematoma expansion based solely on the intraparenchymal hematoma component, which may have underestimated its occurrence in cases with intraventricular extension^29^; and inclusion of only 2 centers, potentially limiting generalisability. Larger multicenter randomised clinical trials adequately powered to test for effect modification, particularly within ultra-early treatment windows, are needed to more robustly assess whether spot sign status can guide individualised BP treatment strategies.

## Conclusion

In patients with ICH within 6 hours of symptom onset, the beneficial association of achieving an intensive SBP target within 60 minutes with hematoma expansion and functional outcome appears to be neither dependent on nor modified by spot sign status. These findings suggest that rapid BP reduction should be pursued in patients with acute ICH regardless of spot sign presence.

## Supplementary Material

aakaf024_RAINSpot_v2_Supplemental_Material

## Data Availability

Further data are available from the authors upon request.
