# Supplementary material for: Influence of spot sign on the association between rapidly achieving blood pressure reduction and intracerebral haemorrhage outcomes
Source: Eur Stroke J. 2026 Jan 1;11(1):aakaf024. doi: 10.1093/esj/aakaf024 (PMC12866633; doi:10.1093/esj/aakaf024)
Supplement: aakaf024_RAINSpot_v2_Supplemental_Material [file aakaf024_rainspot_v2_supplemental_material.pdf]

Influence of Spot Sign on the Association Between Rapidly Achieving Blood Pressure Reduction and Intracerebral Hemorrhage Outcomes

Sousa et al.

SUPPLEMENTAL MATERIAL

Table of Contents:

Supplemental Figure 1. Cohort Flowchart

Supplemental Table 1. Baseline Characteristics of the Patients Included in Hematoma Expansion Analysis Based on the Occurrence of Hematoma Expansion

Supplemental Table 2. Multiple Regression Analyses Examining the Associations of Spot Sign Presence in Any Phase of Multiphase Computed Tomography and Systolic Blood Pressure Target Achievement Within 60 Minutes with Primary and Secondary Outcomes

Supplemental Table 3. Sensitivity Multiple Logistic, Ordinal, and Linear Regression Analyses of the Association Between Arterial Spot Sign and SBP Target ≤60 Minutes With Primary and Secondary Outcomes Further Adjusted for GCS and NIHSS Scores, and Considering Different ICH Expansion Definitions

Supplemental Table 4. Sensitivity Multiple Regression Analyses of the Association Between Arterial Spot Sign and Time From Antihypertensive Bolus to SBP Target (as a Continuous Variable) With Primary and Secondary Outcomes

Supplemental Figure 1. Cohort Flowchart. CTA: computed tomography angiography; mRS: modified Rankin Scale.

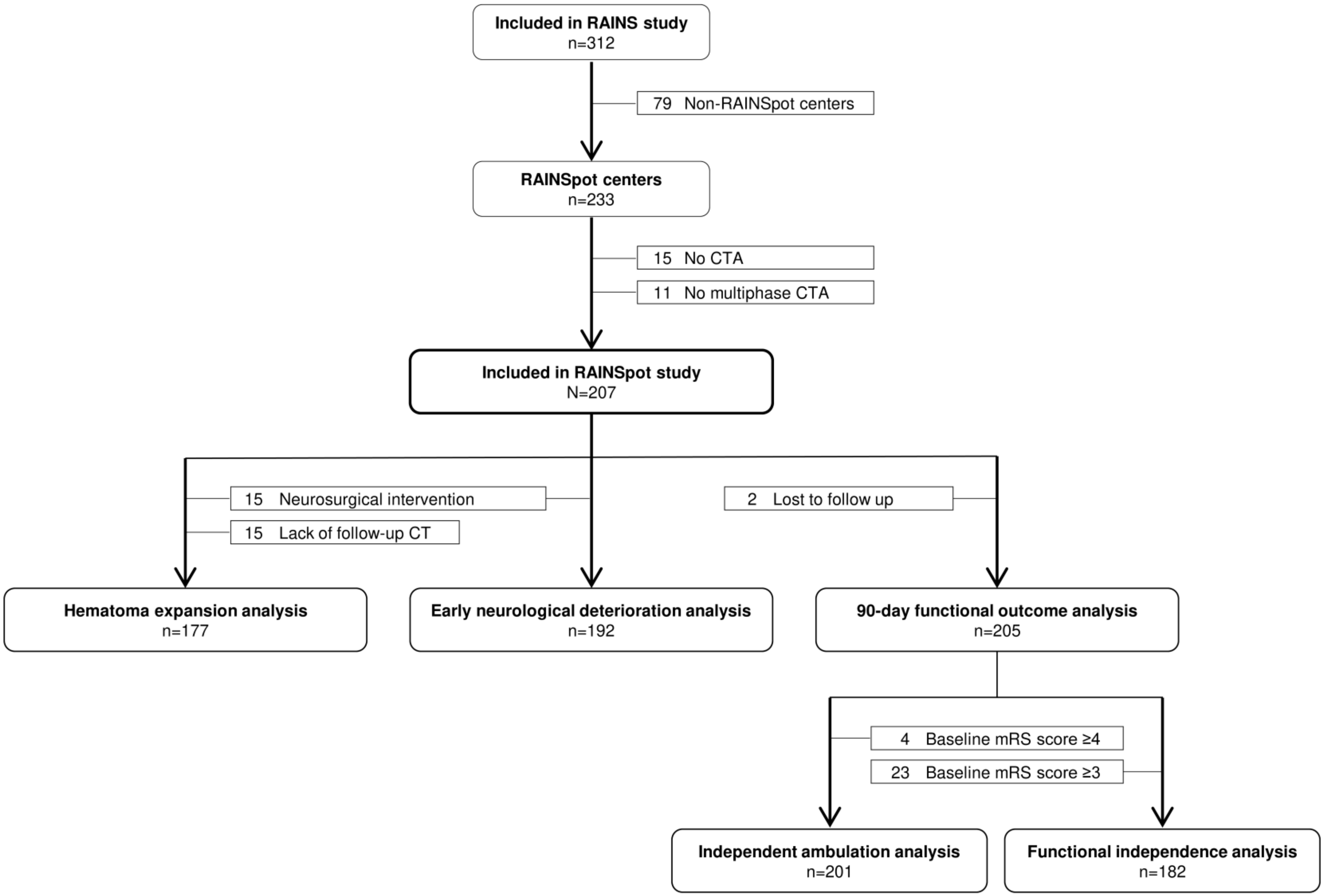

Supplemental Table 1. Baseline Characteristics of the Patients Included in Hematoma Expansion Analysis Based on the Occurrence of Hematoma Expansion

	Patients included in expansion analysis	Hematoma expansion		
	(n=177)	Yes (n=46)	No (n=131)	*p**
**Age, mean±SD, y**	70.5±13.0	74.4±11.1	69.1±13.4	0.014
**Male sex**	114 (64.4)	30 (65.2)	84 (64.1)	0.894
**History of hypertension, n (%)**	139 (78.5)	38 (82.6)	101 (77.1)	0.434
**Antihypertensive drugs use, n (%)**	121 (68.4)	35 (76.1)	86 (65.6)	0.190
**Antiplatelet drugs use, n (%)**	36 (20.3)	11 (23.9)	25 (19.1)	0.484
**Anticoagulant drugs use, n (%)**	25 (14.1)	9 (19.6)	16 (12.2)	0.218
**GCS score, median (p25-p75)**	15 (14-15)	15 (14-15)	15 (14-15)	0.369
**NIHSS score, median (p25-p75)**	12 (6-17)	14 (10-19)	11 (6-16)	0.019
**Systolic BP, mean±SD, mmHg**	177.7±23.4	181.5±24.0	176.3±23.2	0.163
**Diastolic BP, mean±SD, mmHg**	92.3±20.0	92.9±23.3	92.2±18.8	0.981
**Onset-to-imaging time, median (p25-p75), min**	114 (76-202)	99 (72-138)	125 (82-224)	0.012
**ICH volume, median (p25-p75), mL**	10.4 (5.2-25.9)	13.2 (5.1-24.5)	10.3 (5.2-26.9)	0.968
**Lobar ICH location, n (%)**	42 (23.7)	13 (28.3)	29 (22.1)	0.401
**Intraventricular extension, n (%)**	53 (29.9)	10 (21.7)	43 (32.8)	0.158
**Subarachnoid extension, n (%)**	27 (15.3)	8 (17.4)	19 (14.5)	0.639
**Arterial spot sign, n (%)**	56 (31.6)	25 (54.3)	31 (23.7)	<0.001
**Spot sign in any multiphase CTA phase, n (%)**	69 (39.0)	27 (58.7)	42 (32.1)	0.001

BP: blood pressure; GCS: Glasgow coma scale; ICH: intracerebral hemorrhage; NIHSS: National Institutes of Health Stroke Scale.

**p* values determined with Pearson χ^2, Student *t*, or Mann-Whitney *U* tests.

Supplemental Table 2. Multiple Regression Analyses Examining the Associations of Spot Sign Presence in Any Multiphase Computed Tomography Phase and Systolic Blood Pressure Target Achievement Within 60 Minutes with Primary and Secondary Outcomes

	Hematoma expansion	Early neurological deterioration	mRS score at 90 days	90-day independent ambulation	90-day functional independence
**Models without interaction term, aOR (95% CI)**					
**Spot sign in any phase**	2.84 (1.25–6.65)*	2.05 (0.90–4.72)	2.14 (1.22–3.79)*	0.41 (0.18–0.91)*	0.59 (0.25–1.31)
**SBP target ≤60 minutes**	0.28 (0.11–0.65)*	0.61 (0.27–1.41)	0.44 (0.25–0.78)*	4.49 (1.80–12.11)*	3.03 (1.25–7.84)*
**Age (per 1-year increase)**	1.06 (1.02–1.10)*	1.01 (0.97–1.04)	1.05 (1.03–1.08)*	0.92 (0.89–0.96)*	0.98 (0.94–1.01)
**Male sex**	0.66 (0.27–1.54)	0.72 (0.29–1.67)	0.74 (0.42–1.30)	1.48 (0.66–3.44)	1.20 (0.53–2.73)
**Antiplatelets**	1.33 (0.48–3.55)	1.22 (0.43–3.30)	0.74 (0.39–1.43)	3.33 (1.18–10.15)*	0.67 (0.25–1.75)
**Anticoagulants**	1.14 (0.34–3.72)	2.78 (0.96–8.15)	2.79 (1.26–6.33)*	0.22 (0.05–0.79)*	0.27 (0.06–0.98)*
**Baseline SBP (per 10-mmHg increase)**	1.02 (0.85–1.21)	1.10 (0.92–1.32)	1.07 (0.94–1.21)	0.94 (0.78–1.13)	1.08 (0.90–1.29)
**Onset-to-imaging time (per 10-min increase)**	0.92 (0.87–0.97)*	0.94 (0.89–0.99)*	0.97 (0.95–1.00)	1.07 (1.02–1.12)*	1.06 (1.01–1.10)*
**ICH volume (per 10-mL increase)**	0.91 (0.74–1.11)	1.35 (1.15–1.63)*	1.44 (1.25–1.67)*	0.55 (0.40–0.72)*	0.57 (0.41–0.75)*
**Intraventricular extension**	0.29 (0.11–0.72)*	0.96 (0.40–2.22)	2.86 (1.59–5.20)	0.18 (0.07–0.42)*	0.31 (0.12–0.74)*
**Interaction term, *p*†**					
**Spot sign in any phase and SBP target ≤60 minutes**	0.892	0.758	0.254	0.238	0.576

Adjustments were made for age, sex, antiplatelet and anticoagulant use, baseline SBP, onset-to-imaging time, ICH volume, and intraventricular extension at baseline.

aOR: adjusted odds ratio; ICH: intracerebral hemorrhage; mRS: modified Rankin Scale; NIHSS: National Institutes of Health Stroke Scale; SBP: systolic blood pressure.

*$p<0.005$ (Wald test).

†p for interaction values determined with likelihood ratio test.

Supplemental Table 3. Sensitivity Multiple Logistic, Ordinal, and Linear Regression Analyses of the Association Between Arterial Spot Sign and SBP Target ≤60 Minutes With Primary and Secondary Outcomes Further Adjusted for GCS and NIHSS Scores, and Considering Different ICH Expansion Definitions

	**Multiple regression analyses without interaction term***		**Interaction term, *p*†**
	**Arterial spot sign**	**SBP target ≤60 minutes**	
**Further adjusted for GCS and NIHSS scores**			
**Hematoma expansion (>6 mL or >33%)**	3.77 (1.59–9.26)‡	0.26 (0.09–0.65)‡	0.600
**Early neurological deterioration**	2.50 (1.08–5.79)‡	0.55 (0.23–1.28)	0.482
**mRS score at 90 days**	1.96 (1.05–3.68)‡	0.59 (0.33–1.07)	0.188
**Independent ambulation at 90 days**	0.46 (0.17–1.18)	2.31 (0.82–6.90)	0.265
**Functional independence at 90 days**	0.43 (0.15–1.17)	1.61 (0.56–4.82)	0.285
**Considering different ICH expansion definitions**			
**Absolute expansion**	6.44 (2.27–10.61)‡	-4.88 (-8.86–-0.90)‡	0.176
**Absolute expansion >6 mL**	6.34 (2.43–17.82)‡	0.46 (0.17–1.21)	0.591
**Absolute expansion >12.5 mL**	11.12 (2.75–58.36)‡	0.39 (0.11–1.36)	0.210
**Relative expansion**	37.48 (16.70–58.26)‡	-21.75 (-41.61–-1.90)‡	0.227
**Relative expansion >33%**	4.58 (1.73–12.84)‡	0.18 (0.06–0.46)‡	0.304
**Relative expansion >66%**	4.58 (1.41–15.91)‡	0.31 (0.10–0.96)‡	0.707
**Severe hematoma expansion (>12.5 mL or >66%)**	4.61 (1.62–13.95)‡	0.44 (0.16–1.22)‡	0.745

Adjustments were made for age, sex, antiplatelet and anticoagulant use, baseline SBP, onset-to-imaging time, ICH volume, and intraventricular extension at baseline.

GCS: Glasgow coma scale; ICH: intracerebral hemorrhage; mRS: modified Rankin Scale; NIHSS: National Institutes of Health Stroke Scale; SBP: systolic blood pressure.

*Data are expressed as adjusted odds ratios (95% CI) for logistic and ordinal models, and as β coefficients (95% CI) for linear models.

†*p* for interaction values determined with likelihood ratio test.

‡*p*<0.005 (Wald test).

Supplemental Table 4. Sensitivity Multiple Regression Analyses of the Association Between Arterial Spot Sign and Time From Antihypertensive Bolus to SBP Target (as a Continuous Variable) With Primary and Secondary Outcomes

	Multiple regression analyses without interaction term, aOR (95% CI)		Interaction term, *p**
	Arterial spot sign	Time from antihypertensive bolus to SBP target (per 10-min increase)	
**Hematoma expansion**	4.05 (1.73–9.88)†	1.03 (1.01–1.06)†	0.119
**Early neurological deterioration**	2.22 (0.96–5.09)	1.01 (0.98–1.05)	0.617
**mRS score at 90 days**	2.33 (1.27–4.27)†	1.03 (1.01–1.05)†	0.288
**Independent ambulation at 90 days**	0.40 (0.16–0.96)†	0.93 (0.89–0.97)†	0.287
**Functional independence at 90 days**	0.43 (0.16–1.04)	0.95 (0.91–0.99)†	0.330

Adjustments were made for age, sex, antiplatelet and anticoagulant use, baseline SBP, onset-to-imaging time, ICH volume, and intraventricular extension at baseline.

aOR: adjusted odds ratio; mRS: modified Rankin Scale; SBP: systolic blood pressure.

**p* for interaction values determined with likelihood ratio test.

†$p<0.005$ (Wald test).
